# Rethinking psychiatric symptoms: the role of measurement heterogeneity in diagnostic validity

**DOI:** 10.1007/s40656-025-00659-5

**Published:** 2025-03-19

**Authors:** Daniel Montero-Espinoza

**Affiliations:** Institute of Philosophy, Leibniz Universitat Hannover, Lange Laube 32, 30159 Hannover, Lower Saxony Germany

**Keywords:** Diagnostic validity, Measurement heterogeneity, Transdiagnostic research, Anhedonia, Symptoms

## Abstract

The current research environment in psychiatry is marked by the discredit of the main psychiatric classifications. The common narrative about the DSM holds that the current diagnostic categories lack diagnostic validity. This claim is supported by the high degrees of diagnostic heterogeneity and comorbidity among diagnosed patients. Current attempts to overcome these problems emphasize the need to develop alternative ways of investigating psychopathology that no longer rely on the DSM categories. In this line, transdiagnostic research initiatives such as RDoC promote the abandonment of the DSM categories while still relying on traditional psychiatric symptoms. This reliance assumes that symptoms do not pose similar problems to the ones commonly ascribed to the DSM categories. In this article, I challenge what I call the “received view of symptoms” and argue that a closer look at symptom measurement reveals that different measurements of purportedly the same symptom differ from each other in ways that have an impact on both psychiatric research and clinical practice. Furthermore, I show that psychiatric symptoms are not “neutral” vis-à-vis the DSM categories. To illustrate my points, I use a case study from the history of the measurement of anhedonia. Based on this case study, I argue that in addition to diagnostic heterogeneity, there is also symptom measurement heterogeneity. Finally, I suggest a reassessment of the common narrative about the DSM’s lack of diagnostic validity in light of my case study and argue that closer attention should be given to symptoms.

## Introduction

Over the last decades, researchers and clinicians have gradually converged toward a common narrative about the DSM. On the one hand, it is widely recognized that the DSM has improved interrater-reliability (Aragona, 2015; Insel, 2013; Klerman, 1986). This means that based on the diagnostic criteria comprised by a diagnostic category (e.g., bipolar disorder, major depressive disorder) different clinicians consistently arrive at the same diagnosis given a patient with a specific symptom profile. Put more simply, the DSM has become a *lingua franca* for the field. On the other hand, the DSM is often accused of lacking *diagnostic validity* (Kozak & Cuthbert, 2016; Hyman, 2010). In psychiatry, diagnostic validity refers to the accuracy with which a diagnostic category leads to the identification of a homogeneous group of clinical instances of a specific mental disorder, i.e., patients who share the same condition (APA; Rodrigues & Banzato, 2015). Thus, while clinicians may largely agree about patient P being an instance of the diagnostic category D, the diagnostic category D fails to pinpoint a homogeneous group of patients based on the list of diagnostic criteria (i.e., list of symptoms) comprised by such category.

Although criticisms against the DSM vary in severity, they share the view that the current classification system no longer constitutes a promising framework for the development of new clinical treatments. Given that psychiatric taxonomies provide the general framework for the study of psychopathology, researchers find themselves at a crossroads: On the one side, the development of a new classification system requires the creation of a new database that can inform such classification. On the other side, such a database cannot be created as long as research is still conducted on the basis of the old system. The current research landscape in psychiatry is thus driven by the goal of developing alternative ways of investigating and classifying psychopathology that no longer rely on the DSM categories. In this line, some transdiagnostic research initiatives promote the abandonment of the DSM categories and their replacement with cross-diagnostic constructs. However, while advocating for a new taxonomy, transdiagnostic research initiatives still rely on traditional psychiatric symptoms under the assumption that they do not pose similar problems to the ones commonly ascribed to the DSM categories.

In this article, I challenge the *received view of symptoms* readily adopted by some of the most prominent transdiagnostic research initiatives. Such a view assumes that psychiatric symptoms are not influenced by the DSM categories (for an argument challenging this view, see Fellowes, 2017), and also, that the problems ascribed to the DSM categories—in particular, *heterogeneity*—are not analogously replicated across symptoms. I rely on a case study from the history of the measurement of anhedonia to show that both assumptions are mistaken. Towards the end, I reassess the common narrative about the DSM’s lack of diagnostic validity in light of my case study and suggest that *symptom measurement heterogeneity* might play a role in the DSM’s lack of diagnostic validity. Finally, I elucidate under what circumstances such heterogeneity becomes potentially detrimental.

## Diagnostic validity in the DSM-III

In 1980, the third edition of the DSM-III was launched with the goal of providing an evidence-based diagnostic system for evaluating psychiatric patients. The overreliance of psychiatry on psychodynamic theory prior to the DSM-III had led to diagnostic imprecision and inadequate assessment of symptoms (Andreasen, 2007; First, 2012; Harrington, 2019). To amend these issues, the DSM-III focused on increasing inter-rater reliability by establishing a common vocabulary that included diagnostic criteria from an ‘atheoretical perspective’, i.e., without referring to hypothetical etiologies (see Klerman et al., 1984, and Cooper, 2004 for critical comments). In addition to reliability, the task force responsible for the DSM-III was aware of the importance of improving the diagnostic validity of all diagnostic categories. However, due to the lack of information about potential validators, *validation* was regarded as a later step in the development of an evidence-based psychiatric nosology; a step that, the task force hoped, would follow from successfully increasing reliability (Aragona, 2015). This view was influenced by the 1970 article by Robins & Guze, where they suggested a few methods for determining whether a particular psychiatric disorder could be considered valid. Some of these methods were familial aggregation, characteristic longitudinal course, response to treatment, and laboratory tests (Robins & Guze, 1970). Eventually, the reliability of psychiatric diagnoses improved, but diagnostic validity remained an elusive goal. Over the following years, the DSM-III and its successors became ‘universally and uncritically accepted as the ultimate authority on psychopathology and diagnosis’ and the gold standard for training residents and undergraduates in the US (Andersen 2007, p. 111). While evidence supporting the diagnostic validity of the DSM categories lagged, early reports of its increased reliability were widely used to promote the DSM worldwide (Aragona, 2015, p. 41). The popularity of the DSM outgrew the efforts to implement the validation methods outlined by Robins and Guze. The diagnostic criteria initially intended to guide diagnostic practice by describing common symptoms among certain psychiatric diagnoses also facilitated investigative exchange, but at the expense of relying on understudied psychiatric constructs.

### Comorbidity and heterogeneity

The DSM’s lack of diagnostic validity manifests as two pressing problems that affect both psychiatric research and clinical practice. First, most diagnostic categories exhibit a considerable degree of overlap between their diagnostic criteria. One direct consequence of this overlap is the high *comorbidity*^1^ rate between different diagnostic categories, i.e., for any given patient, the chance that they cross the diagnostic threshold of more than one disorder is high. Indeed, around 45% of all diagnosed patients fulfill the diagnostic criteria of more than one DSM category, and in patients with certain diagnoses, the occurrence of multiple diagnoses is more common than a single diagnosis (Bueter, 2019, p. 7). The second problem arises from how diagnostic categories are organized. From the third edition onwards, DSM categories are operationalized in terms of clusters of symptoms. Each category comprises a set of diagnostic criteria (i.e., symptom descriptions) that allegedly identify instances of the same disorder. For instance, in the DSM-5, the diagnostic category *Major Depressive Disorder* (MDD) comprises a list of nine diagnostic criteria plus a minimum time frame of symptom manifestation. Diagnostic categories are polythetic. This means that to be diagnosed with MDD, for instance, a patient must experience a minimum of five symptoms (including the two core symptoms of MDD) for a minimum period of two consecutive weeks. Thus, in practice, two patients can be diagnosed with MDD while having only two symptoms in common. The polythetic character of the current diagnostic categories allows for substantial variability of symptom profiles among patients with the same diagnosis. According to Fried and Nesse (2015, p. 97), there are at least 227 unique symptom profiles that cross the diagnostic threshold of MDD as outlined in the DSM-5. This is known as *diagnostic heterogeneity* (Allsop et al., 2019). The high rates of comorbidity and heterogeneity in clinical practice are often seen as strong indicators of the DSM’s general lack of diagnostic validity. Furthermore, they are often cited as one of the reasons why the average efficacy of marketed psychiatric drugs in the US is low (about 50% according to a study published by Wong et al., 2010).

### The rise of transdiagnostic research

In 2010, Steven Hyman—a former director of the NIMH—famously stated that thanks to its improvement of reliability, the DSM has become a *lingua franca* on which most psychiatry studies have relied in the past decades. This reliance, however, has led to the premature *reification* of the DSM categories, i.e., despite their low diagnostic validity, these categories were increasingly treated as referring to distinct disease entities that exist with independence of the observer’s conceptualization (for a discussion on why researchers essentialize mental disorders, see Adriaens & De Block, 2013). In Hyman’s words, the DSM created an unintended “epistemic prison” (Hyman, 2010, p. 157) that controlled the research agenda by determining the questions that researchers could ask as well as the means devised to provide answers to those questions.

In this context, a growing number of research initiatives in psychiatry began to develop alternative frameworks to investigate psychopathological phenomena beyond the constraints of the DSM categories. Transdiagnostic research thus emerged with a focus on understanding and treating aspects of mental disorders that were considered common across different diagnostic categories. The long-term goal was to identify and validate promising targets for clinical intervention at different levels of analysis and thus reduce the high rates of comorbidity and heterogeneity associated with the DSM. If the heterogeneity of the diagnostic categories was partly causing the low response to medication, then, the obvious solution was to cluster patients on the basis of categories that were more fine-grained than the DSM categories. Aligning with the emerging movement of precision psychiatry, some transdiagnostic research initiatives—most notably RDoC—aim to revamp the taxonomic landscape in psychiatry by integrating data from different levels of analysis such as genes, neurocircuitry, and behavior (Cuthbert & Insel, 2013). However, this is not necessarily the approach taken by all transdiagnostic research initiatives.

A recent systematic review of transdiagnostic psychiatry revealed that the term “transdiagnostic” is used in a rather loose and unstandardized way to refer to clinical studies and research approaches focusing on variables that cut across the boundaries of traditional DSM categories (Fusar-Poli et al., 2019). Although they do not constitute a unified program, transdiagnostic research initiatives share the tenet that the risk and maintenance factors and processes involved in the manifestation of psychiatric symptoms show no specificity for particular diagnostic categories (Dalgleish et al., 2020, p. 184); instead, such factors and processes appear as cutting across the boundaries of the DSM categories. Thus, a typical transdiagnostic study might compare different DSM categories in order to assess their diagnostic boundaries and cross-cutting features (Fusar-Poli et al., 2019). Alternatively, a study might focus on a single transdiagnostic construct (e.g., sleep patterns) and analyze its variation across a sample comprising individuals with different DSM diagnoses. Importantly, a common feature of all transdiagnostic studies is the use of samples where two or more DSM categories are represented (as opposed to the more traditional single-diagnosis studies).

### The received view of psychiatric symptoms

In transdiagnostic research, psychiatric symptoms are seen and treated as “transdiagnostic in nature”, i.e., as cutting across traditional diagnostic boundaries. To a good extent, this is an obvious implication of the problem of comorbidity. In the DSM-5, for instance, symptoms such as *anhedonia* and *insomnia* are included in the diagnostic criteria of more than one diagnostic category. The pervasiveness of this symptom-overlap is what best explains the high rates of comorbidity among diagnosed patients. From a transdiagnostic perspective, it thus makes sense to get rid of the DSM categories but keep the symptoms. Comparted to the DSM categories, symptoms correspond to relatively specific patient experiences or behaviors that can be more straightforwardly measured or addressed. They seem to provide a more granular and flexible framework for understanding mental disorders; one that, apparently, does not inherit the variability that the polythetic structure of the DSM categories entails. The idea that psychiatric symptoms cut across several diagnostic categories is not a novel element of current transdiagnostic approaches; rather, it is a *received view* whose implications have gone largely unassessed.

In a recent article, Fellowes (2021) notes that while critics of the DSM promote the abandonment and subsequent replacement of the DSM categories with a new taxonomy, they seem much less concerned about symptoms (which are the very “constituents” of the syndromes denoted by such categories). For instance, in an article titled “Drop the language of disorder”, Kinderman et al. (2013) advocated for the abandonment of the DSM categories while still talking of traditional psychiatric symptoms (e.g., low mood and auditory hallucinations) as study objects that future research should focus on. In a similar vein, Bruce Cuthbert–former director of the RDoC division at the NIMH- declared that “the concern about the current diagnostic environment has not been so much with the symptoms themselves” (Cuthbert, 2014, p. 32), and later, that “it is the grouping of symptoms into what have turned out to be overly heterogeneous syndromes that poses the problems for research” (Cuthbert, 2015, p. 94). While projects like RDoC hope to revamp the diagnostic landscape by replacing the current diagnostic categories with psychobiological dimensions, nearly no mention is made in its many publications as to whether psychiatric symptoms need any replacement (Fellowes, 2021, p. 4503). The tacit assumption that operates in the received view is that symptoms are “DSM-free”, i.e., they do not depend on considerations made on the basis of the diagnostic categories. Second, the received view assumes that symptoms do not play a role in the lack of diagnostic validity of the DSM categories.

In this article, I argue that a closer look at *symptom measurement* reveals that the view on psychiatric symptoms readily adopted by some of the current transdiagnostic approaches is unwarranted. In particular, I claim that different measurements of purportedly the same symptom differ from each other in ways that might have an impact on both psychiatric research and clinical practice. To support this claim, I use existing scales for the measurement of anhedonia as a case study to show that the same individual can, in principle, qualify as experiencing anhedonia or not depending on the scale with which their subjective experience is assessed. This, I suggest, generates a type of heterogeneity comparable to the diagnostic heterogeneity caused by the polythetic structure of the DSM categories. Later, I rely on the same case study to show that one of the measures of anhedonia (the Chapman Scales for Physical and Social Anhedonia) was deliberately developed with the aim of “eliminat[ing] the effects of depression” on the scale, i.e., the scale was developed with the explicit goal of excluding what was regarded by some researchers as a “depressive type” of anhedonia (Chapman et al., 1976, p. 376). Thus, while symptoms such as anhedonia can in principle be found in more than one diagnostic category in the DSM, some of the instruments used for its assessment were developed and calibrated targeting specific DSM categories. This, I contend, challenges the view that symptoms are “neutral” or independent from specific DSM disorders. Moreover, to the extent that transdiagnostic approaches such as RDoC rely *de facto* on symptom constructs and measures that are not DSM-free, they fail to accomplish their goal of “free[ing] research from constraint by current diagnostic entities” (Sanislow et al., 2010, p. 637). In the last section, I reexamine the common narrative about the DSM’s lack of diagnostic validity in light of the insights derived from my case study.

## What is a symptom?

Before taking a closer look at symptom measurement in psychiatry, some conceptual housekeeping is necessary. In the following subsections, I provide a brief overview of what are symptoms in general medicine, and later, what are the specificities of psychiatric symptoms. First, I flesh out the view that symptoms, unlike mere experiences, are inherently indicative and this constitutes its core trait. On this basis, I specify how the notion of symptom relates to the notions of *syndrome* and *sign* according to medical semiology. In subsection three, I introduce Fellowes’ philosophical account of psychiatric symptoms according to which symptoms must be seen as *phenomena* in Bogen and Woodward’s (1988) sense. In the last subsection, I expand on Fellowes’ account by elaborating on the role that symptom measurements play in data-to-phenomena reasoning and suggest that symptoms may give rise to problems similar to the ones commonly attributed to the DSM categories.

### Symptoms as indicators

In most branches of medicine, clinical encounters normally start with a person describing a discomforting or unusual experience (e.g., a burning discomfort felt most intensely behind the breast bone). The clinician then translates such description into a symptom: chest pain. Far from mere paraphrasing, this translation plays the key role of ascribing certain dispositions and a potential etiology to the person’s experience (Wardrope & Reuber, 2022, p. 395). Symptoms, unlike “raw” experiences and behaviors, are traditionally considered pointers or indicators of diseases, i.e., symptoms are always symptoms *of* an illness. By assessing several symptoms in patients, medical practitioners determine a symptom profile based on which they seek to deliver a precise diagnosis and later determine the course of treatment. In this context, the utility of a symptom is relative to its specificity; the fewer diseases a symptom is indicative of, the more informative and useful it is for diagnosing a condition. Conversely, if a symptom is common to many diseases, it becomes less helpful in pinpointing a specific diagnosis. As mentioned earlier, in psychiatry, the indicativeness of symptoms is highly unspecific. Most psychiatric symptoms appear in the diagnostic criteria of more than one DSM category which renders them poor guides for differential clinical diagnosis and explain the high rates of comorbidity among psychiatric patients.

In psychiatry, unlike in many other branches of medicine, the etiology (i.e., the set of causes) of mental disorders and their symptoms is largely unknown. From its third edition onwards, the DSM contains almost no reference to the potential causal mechanisms that lead to the manifestation of psychiatric symptoms. In the lack of a clear etiology, the approach taken by the DSM is to organize psychiatric symptoms into “theoretically meaningful clusters of symptoms” or *syndromes* (Wilshire et al., 2021, p. 323). A syndrome is a set of symptoms that occur together with certain regularity across individuals and *suggest* the presence of a disease. Symptoms such as low mood, for instance, are more likely to be accompanied by anhedonia than by worrying thoughts or verbosity (ibid.). Thus, each DSM category constitutes a meaningful cluster insofar as it comprises a list of symptoms that in practice occur together with a relatively high frequency, which in turn, suggests that they share a set of common causes (or even just one cause), whatever these are.

### Signs and symptoms

In somatic medicine, it is common to distinguish between *signs* and *symptoms* of diseases. The former are defined as observable indicators of the presence of a disease (e.g., frequent urination as a sign of diabetes). Symptoms, on the other hand, are self-reported subjective experiences that cannot be directly observed by a third person (e.g., feeling very thirsty as a symptom of diabetes). In psychiatry, however, this distinction is somewhat blurred and often omitted (Harrison et al., 2017). In the DSM-5, in particular, signs and symptoms appear undifferentiated in the diagnostic criteria of most diagnostic categories. For instance, the diagnostic criteria of MDD in the DSM-5 include ‘significant weight loss or weight gain’ and ‘indecisiveness’, a sign and a symptom respectively. One explanation for the lack of a sharp distinction between the two is that often symptoms are not as immediately evident to those who have them as, say, an unquenchable thirst. In those cases, symptom measures (e.g., rating scales) often include items referring to behavioral patterns. Individuals experiencing irritability, for instance, might show a lack of insight regarding their state of mind. Nonetheless, irritability can *manifest* in terms of relatively specific behavioral patterns such as acting out violently in a context where such acting is not justified. Thus, instances of mental disorders are identified on the basis of diagnostic criteria that comprise signs and symptoms, and specific symptoms, in turn, can be assessed through the use of self-report questionnaires that comprise behavior-based questions as a means to gain insight into an experience that may not be immediately apparent to their bearers. For instance, ‘indecisiveness’—a symptom of MDD—is measured by the ‘Indecisiveness Scale’ developed by Germeijs and Boeck (2002) which includes items of a more behavioral kind such as “I tend to leave decisions to someone else”.

### Symptoms as phenomena

It is useful to distinguish here between two steps associated with the development and clinical use of symptom measures. One of these steps corresponds to the clinical practice of identifying instances of a symptom (and assessing their severity) in patients through the use of measures such as self-report questionnaires and rating scales. Prior to the use of a symptom measure within a clinical context, the first step involves conceptualizing a symptom and developing a way of measuring it, which, in turn, presupposes a symptom construct. The conceptualization of a symptom, be it a first-person experience or a behavioral pattern, requires the agreement of researchers upon some features of the symptom as belonging to its hypothetical core attributes. This need for agreement on a symptom’s core attributes highlights the constructed nature of symptoms as concepts rather than straightforward things. In line with this, Fellowes argues in a recent article that individuals do not straightforwardly *have* symptoms; instead, he claims, *it is more accurate to say that people behave in certain ways but are assigned symptoms*^2^ (2021, p. 4504). In this view, symptoms constitute general conceptual categories under which the experiences or behaviors of patients may fall. Within the clinical context, an individual’s particular experience can then constitute an *instance* of a symptom, but not the symptom itself. In our previous example, the discomforting experience described by a patient at the clinical encounter constitutes an instance of chest pain; as such, it contains a great number of details that are unique to their particular experience. Some of those details might be captured by the general concept of the symptom “chest pain”, but most of them will not. This is because the symptom “chest pain” is conceptualized in a rather abstract and highly generalizable manner. This entails abstracting away many of the details present in individual instances of a first-person experience or behavior. In the DSM-5, for instance, “stereotyped or repetitive motor movements” is a symptom of autism spectrum disorder (ASD). Specific instances of behavior such as lining up toys or flipping objects repeatedly contain innumerable details that are not retained in the general concept of the symptom. Details such as the exact length of the line of toys or the color choice of the toys are deemed irrelevant for determining whether an instance of behavior is an instance of the symptom at stake and therefore will be not included in the concept of stereotyped or repetitive motor movements. Both examples illustrate Fellowes’ broader point that symptoms are not straightforward descriptions of experience or behavior but are constructed through a process of abstraction that involves discarding many of the details present in individual cases.

Drawing upon the distinction between *data* and *phenomena* introduced by Bogen and Woodward’s 1988 seminal paper, Fellowes argues that we should conceive symptoms as phenomena and instances of behavior as data (2021, p. 4499). As such, symptoms then constitute unobservable yet measurable regularities whose attributes can be inferred from instances of behavior. In his view, symptoms—as phenomena—are not simply “ready-made parts of the world” to be discovered via empirical means; rather, they are “constructed from behavior” in a process that involves making decisions over the attributes that will be included in the general concept of a symptom (ibid, p. 4507). This idea departs from the more realist view of phenomena found in the 1988 paper, and relies on Woodward’s later account of phenomena where he argues that “decisions to ignore or discard data play a central role in virtually all data-to-phenomena reasoning” (Woodward, 2000, p. 177 as in Fellowes, 2021, p. 4506). In psychiatry, such kinds of decisions are influenced by prior theoretical understandings of symptoms. Fellowes illustrates this point by showing how different theories of autism throughout the second half of the twentieth century determined the hypothesized core attributes of the symptom “low social skills”. From the 1940s to the 1960s, with psychoanalysis as a backdrop, psychiatrists gave emotional processes a prominent role in accounting for the etiology of the symptoms of autism, including “low social skills”. Around the 1960s up until the 1980s, with influential psychiatrists such as Aaron Beck standing for the cognitive turn in psychology, an increasing focus on cognitive processes replaced the previous interest in emotional processes. Consequently, says Fellowes, the symptom “low social skills” in autism became reconceptualized in terms of cognitive deviances (e.g., theory of mind deficits) instead of as the result of reactions to inner emotional processes (2021, p. 4508).

### Symptoms and their measurements

Fellowes’ view is right in pointing out the theory-ladenness of symptom concepts and the influence that prior theoretical understandings might have on data selection processes. However, one aspect that remains unaddressed in his article is the role that different measurements play in data-to-phenomena reasoning in psychiatric research. Expanding on Fellowes’ view, I argue (1) that a closer look at symptom measurement reveals that different measures of the same symptom often presuppose constructs that differ from each other in clinically relevant ways (i.e., different instruments work with different assumptions about what *is* the thing they intend to measure). These inter-measurement differences need not be driven by vastly different theories about the etiology of the symptom at stake. Rather, those differences take the form of diverging working assumptions embedded in the constructs presupposed by different instruments that aim to measure the same thing. To be sure, those working assumptions diverge with regard to what exactly *is* the thing being measured. This divergence is certainly not always detrimental or unjustified. However, as I later show in this article, it can be so. Under certain circumstances such divergence gives rise to a type of heterogeneity that is comparable to the diagnostic heterogeneity commonly attributed to the DSM categories. A central goal of this article is to shed some light on the circumstances under which such divergence becomes problematic.

Here I distinguish between the notions of *symptom concept* and *symptom construct*. I take the former to be a definition of a symptom (i.e., a linguistic entity) such as the ones found in the DSM or in any psychiatry textbook. The latter is relative to a specific measurement tool and corresponds to the construct of a symptom that is presupposed by such a measurement tool. As I show in the following sections, in some cases having a one-to-many relationship between a symptom concept and the symptom constructs presupposed by different measurement tools aiming to learn about the same phenomenon can be problematic. This may happen, for instance, when the data gathered with a specific symptom measure is used to support claims about a symptom in general in ways that neglect potentially relevant contextual and purpose-related determinants. Importantly, my contention does not presuppose what Alexandrova (2017) refers to as an all-things-considered view of concepts. Such a view would presume that at any given time there is an all-encompassing concept of a symptom relative to which alternative conceptualizations of the same symptom are either partial or wrong. This view would also presuppose that there is a unified general theory underpinning the choices made in articulating such an all-encompassing symptom concept. For the purpose of this article, it suffices to assume that a symptom concept can be informed by several measurement tools in a context-negligent way. This allows, for instance, to work with a view of symptom concepts as *Ballung* concepts because all it matters is that a symptom concept (regardless how partial or specific) can be linked to two or more measures in a problematic way. The extent to which such a link is problematic, on the other hand, is context-dependent. I elaborate on what this means in section five.

Furthermore, I argue that (2) at least some of the instruments currently used for assessing clinical psychiatric symptoms have been devised with the aim of discriminating between individuals diagnosed with different DSM categories. This implies that symptoms might not be as DSM-free as transdiagnostic research assumes. To illustrate (1) and (2), I compare two different rating scales of the symptom *anhedonia*, defined as “the inability to enjoy experiences or activities that normally would be pleasurable” (APA). This comparison shows that the constructs the two scales presuppose in their measurements differ in potentially clinically relevant ways, leaving open the question of whether and to what extent the two scales measure the same thing.

## Anhedonia: a case-study

The concept of anhedonia became widespread in Western psychiatry after its introduction in the DSM-III in 1980 as a necessary criterion of melancholia (Berrios, 1996, p. 344). There, it was defined as a “lack of interest or pleasure in all or almost all usual activities” (APA 1980). Today, anhedonia is considered a symptom of several diagnostic categories (e.g., major depressive disorder, schizophrenia, substance dependence). Despite major developments and changes concerning those diagnostic categories, the *concept* of anhedonia has remained practically unmodified since the DSM-III. The current stability of the concept of anhedonia, however, contrasts with its intricate pre-DSM historical development and the heterogeneity of the measurements currently used for its clinical assessment.

In his *History of Mental Symptoms* (1996), German Berrios provides a historical account of the development of the concept of anhedonia and the behaviors associated with it. His historical analysis shows that the boundaries of “anhedonie” have been fuzzy ever since the term was coined by Theodule Ribot in his 1897’s work on the psychology of sentiments. According to Olivares and Berrios (1995, p. 454), the concept of anhedonia is prone to have fuzzy boundaries because it is negatively defined as the “lack and/or decreased capacity to experience pleasure”, and thus, it heavily relies on the ever-changing concept of pleasure. From a phenomenological perspective, it is unclear whether pleasure constitutes a specific *sensation* or rather a *general quality* attachable to any state of consciousness (Berrios, 1995, p. 455). Relatedly, it is unclear whether there is something fundamentally common to all pleasurable experiences. Throughout the twentieth-century anhedonia was thought of as involving an “inability to *experience* pleasure” (where “pleasure” is seen as a *sensation*), an “inability to be *conscious* about pleasure”, an “inability to *express* the feeling of pleasure”, a “lack of interest”, a “lack of desire or drive”, etc. More recently, much discussion in the research context has revolved around two related issues. First, whether anhedonia encompasses two or more separable yet tightly linked phenomena (i.e., *consummatory*, *anticipatory*, and remembered anhedonias) or whether anhedonia is best depicted as a multidimensional yet unitary phenomenon that includes physical, emotional, and social domains (Gago & García-Blanco, 2015; Ho & Sommers, 2013; Strauss & Gold, 2012; Serretti, 2023). Second, whether anhedonia constitutes a transient phenomenon or a more trait-like long-lasting one. In what follows, I look with some detail at an example of the latter type of issue.

Although the term took root in the continental psychoanalytic tradition much earlier, it wasn’t until the seventies that the concept of anhedonia became widespread in English-speaking psychiatry, and soon after that, the first attempts to develop measures of the condition were made (Berrios & Olivares, 1995, p. 454). Among such attempts, the Scales for Physical and Social anhedonia were developed as early as 1976 by Chapman et al., and remain until nowadays one of the most widely used assessment tools of anhedonia. At the time the Chapman Scales were developed, anhedonia had long been studied as a symptom of both depression and schizophrenia by a number of researchers whose views became quickly a medical standard in the field (Berrios & Olivares, 1995). Both depression and schizophrenia were by then two of the most prominent diagnostic categories of the DSM-II. Four years later, in 1980, the DSM-III legitimated anhedonia as a symptom of the two conditions (Berrios, 1996, p. 342).

In developing their instrument, Chapman et al. tested the initial set of items included in the scales using a sample of 371 college students (Gago & García-Blanco, 2015, p. 3; Chapman et al., 1976, p. 376). The scales comprised a number of self-report true-false items divided into 40 items assessing physical anhedonia and 48 items assessing social anhedonia. The decision to distinguish between the social and the physical domains of anhedonia was made following a prior distinction established by the authors between “physical pleasures” (i.e., sensorial pleasures such as eating), “interpersonal pleasures” (i.e., pleasures of interacting with people) and finally “other pleasures”, the latter of which was omitted from their scales “in the interest of time” (ibid.).

In their seminal paper, Chapman et al. stated that their research on anhedonia was stimulated by Paul Meehl’s 1973 book *Psychodiagnosis* where “reduced hedonic capacity” was hypothesized as a genetically heritable trait of all schizophrenic patients (Chapman et al., 1976, pp. 374–375). This hypothesis, however, did not extend to anhedonia in depressive patients who were seen as exhibiting a much less “characterological” type of anhedonia. Accordingly, the Chapman Scales for Physical and Social Anhedonia were deliberately developed with the explicit goal of “elminat[ing] the effects of depression” on the scales (Chapman et al., 1976, p. 376). The rationale for this decision was that, unlike the anhedonia found in patients with schizophrenia, the “anhedonia in depression is relatively transient in most depressed patients” (ibid.). In their words: “the anhedonia which we wish to measure is a life-long characterological defect in the ability to experience pleasure” (ibid.). That was the type of anhedonia they believed was exhibited by schizophrenic patients only. The items comprised by the scales were thus “worded to refer to long-standing characteristics rather than to present characteristics” (ibid). For instance, social and physical items included in the Chapman Scales of Anhedonia are respectively: “Getting together with old friends has been one of my greatest pleasures” and “I have always had a number of favorite foods” (ibid).

The construct presupposed by the Chapman Scales of Anhedonia contrasts, for instance, with the one presupposed by the Snaith-Hamilton Pleasure Scale. Together with the Chapman Scales, the Snaith-Hamilton Scale is nowadays considered one of the gold standards for the assessment of anhedonia in psychiatry. Developed by Snaith et al. in 1995, it consists of 14 self-report items to which individuals must respond either “agree” or “disagree”. To determine the set of items, Snaith et al. asked 100 members of the general public to submit a list of five situations that caused them pleasure (1995, p. 99). The goal was to obtain a representative sample of items across several domains such as social interaction, food and drink, sensory experiences, achievement, and pastimes (ibid.). No declared assumption or distinction was made concerning whether the construct they intended to measure was of a transient or rather of a trait-like nature. Nor do they mention whether the type of anhedonia they intended to capture with their scale was distinctive of either schizophrenia or depression (or any other mental disorder). Compared to the Chapman Scales, the items of the Snaith-Hamilton Scale are much less trait-oriented; that is, they do not describe types of behaviors that persist over time. Instead, they convey more punctual and present-time types of situations. Some of the items included are “I would be able to enjoy my favorite meal” and “I would enjoy looking smart when I have made an effort with my appearance” (Snaith et al., 1995, p. 99).

My previous example provides evidence for the claim that, contrary to the received view in transdiagnostic psychiatry, symptom assessment tools are not neutral vis-à-vis the DSM categories. Symptom measures are sometimes developed with the explicit goal of discriminating between different diagnostic groups. The construct presupposed by the Chapman Scales of Anhedonia comprises features that allow the identification of instances of a diagnostic-specific type of anhedonia. Furthermore, it shows that the means by which we assess and gain access to psychiatric symptoms (i.e., *qua* phenomena) differ from each other in potentially relevant ways. Again, this challenges the idea that it is the grouping of symptoms (i.e., the syndromes) that poses a problem for psychiatric research, and not the symptoms themselves (or their measures). In what follows I use the term “symptom measurement heterogeneity” to refer to the divergence among the constructs presupposed by different measures of purportedly the same symptom. The purpose of introducing the term is to establish a parallel with the notion of *diagnostic heterogeneity*, ubiquitous in the psychiatry literature and often mentioned as an indicator of the DSM’s lack of diagnostic validity. According to the thesis of *diagnostic heterogeneity*, the polythetic structure of the DSM categories enables the proliferation of individuals carrying the same diagnostic label while exhibiting markedly heterogeneous symptom profiles. Comparably, *symptom measurement heterogeneity* holds that the heterogeneity of the constructs presupposed by different measures of purportedly the same symptom gives rise to datasets that don’t mean the same things, which can be relevant in contexts where measures are involved. This happens most evidently if, for instance, a patient (or a group of patients) appears as having anhedonia or not depending on what scale is used.

So far, I have argued that current symptom measurement practices give us reason to question some of the tenets held by transdiagnostic psychiatry regarding symptoms. In particular, I have shown that the constructs presupposed by some symptom measures of anhedonia are infused with the DSM categories. I also argued that the current landscape of symptom measurement practices supports the idea that, in addition to diagnostic heterogeneity, there is symptom measurement heterogeneity. In the next section, I first consider some of the challenges that such heterogeneity might pose for psychiatric research and clinical practice by looking at studies on measurement in depression research. Later, I examine why symptom measurement heterogeneity is *not* inherently problematic; bur rather, a justifiable and ineliminable trait of scientific disciplines dealing with complex study-objects. By taking issue with such consideration, I aim to elucidate under what circumstances is symptom measurement heterogeneity potentially detrimental.

## Symptom measurement heterogeneity examined

Admittedly, the acknowledgment of measurement heterogeneity in psychiatry, and possibly in any scientific discipline, is little more than a truism. There is a trivial sense in which there are no two identical measurements. On the other hand, there is no principled criterion to determine the exact point at which two measures of purportedly the same thing are different enough to justify the claim that they no longer measure the same thing. Notwithstanding, we can meaningfully engage with the task of assessing such kind of heterogeneity in terms of its risks and possible effects on research and, in the case of psychiatry, clinical practice. In what follows, I briefly turn to this consideration. First, I look at two studies from the psychiatry literature that take issue with the problems posed by the heterogeneity of measurements in mental health. Together with outlining those problems, I show that even when measurement heterogeneity is thematized as a problem for psychiatric research and clinical practice, the received view of symptoms keeps operating in the background. In the psychiatry literature, the concern about measurement heterogeneity is a concern restricted to psychiatric syndromes. Symptoms are still largely regarded as unproblematic and straightforward things.

### Measurement heterogeneity: lessons from depression research

In Fellowes’ account, we observed that prior theoretical understandings of symptoms play a crucial role in the formation of symptom concepts. In turn, symptom concepts provide the general frame for developing measurement instruments, i.e., they give researchers and clinicians a streamlined idea of the kinds of behaviors they should be looking at. Instruments, on the other hand, presuppose constructs that differ from one another in that they work with varying assumptions about what exactly *is* the thing they aim to measure. Based on the data gathered by such instruments, researchers infer new things about the symptom at stake in each case. In an ideal scenario, researchers would then update and refine their concepts in concordance with the new information. But what if the constructs presupposed by different measurements aiming to capture the same symptom diverge significantly? Relatedly, what if the datasets based on which researchers update their taxonomies and symptom concepts—for instance, in the DSM revisions—do not mean the same things?

In the last decades, researchers have begun to consider the impact that the current abundance of measures of *depression* (i.e., a syndrome) might have on research and clinical practice. In a first-of-its-kind analysis published in 2006, Santor et al. found that since 1918 more than 280 measures of depression have been developed and published (p. 137). Most of them were developed in the 1980s and 1990s, after the publication of the DSM-III (p.143). Their results underscored the high degree of variability among the specific set of symptoms assessed by each scale, the frequency with which symptoms of depression were assessed across different scales, and the number of items dedicated to assessing specific symptoms. The conclusion was unambiguous: different measures of depression differed with respect to which symptoms make up the syndrome of *depression* and also what is the relative salience of different symptoms within the syndrome. About a decade later in a similar study, Fried (2017) analyzed the degree of overlap between the symptoms assessed by the seven most common scales of depression. Fried found that the seven scales encompassed a total of 52 symptoms and, on average, a symptom featured across only three scales. Furthermore, about 40% of the symptoms appeared in a single scale and only 12% of the symptoms appeared across all seven scales (p. 191).

Both studies coincided in their remarks on the difficulties that measurement heterogeneity poses for research and clinical practice. The degree to which different scales of depression differ regarding the set of symptoms they assess jeopardizes the *generalizability* of the results obtained with any particular scale. For instance, a clinical study could demonstrate the effectiveness of an antidepressant medication on a specific scale while, on various other scales, the participants of the study might not exhibit any clinical progress. A further issue, notes Fried, is that the routine practice in depression research is to assess depression with one particular depression scale and draw conclusions about depression in general (2017, p. 191). Furthermore, the rationale for using a specific scale of depression in a study is rarely stated (ibid.). The tacit assumption of this practice seems to be that measures of depression are largely interchangeable, which is highly contestable.

### Contextualizing symptom measurement heterogeneity

Although the previous findings are taken from research on the measurement of depression—which is a syndrome, not a symptom—it is likely that similar issues take place across symptom measurement. In fact, my case study on anhedonia provides some evidence that there is symptom measurement heterogeneity in psychiatry. The impact that such heterogeneity may have on diagnosis and clinical treatment likely depends on the degree of heterogeneity among different measures of purportedly the same thing, but also, on how preponderant is a symptom for a differential diagnosis. If, for example, what is at stake is the core symptom of a syndrome, then, having two symptom measures that greatly diverge from each other might be deciding in the process of diagnosing an individual. Indeed, not all the symptoms exhibited by a patient “weigh” the same when a clinician makes a diagnosis. In some cases, the presence or absence of a symptom may tip the scales toward different diagnoses. The variability introduced by symptom measurement heterogeneity leads us to ponder how much of the DSM’s lack of diagnostic *validity* derives from such heterogeneity. At the outset of this article, I mentioned that diagnostic validity refers to the accuracy with which a diagnostic category (e.g., major depressive disorder) leads to the proper identification of a group of individuals who instantiate the same mental disorder. The common narrative about the DSM holds that it is the grouping of symptoms (and not the symptoms themselves) that poses problems for research and clinical practice. I would like to resist that claim by pointing out that, perhaps, the fact that the DSM fails to pinpoint homogeneous groups of patients based on meaningful clusters of symptoms might sometimes be related to how the instruments used for assessing psychiatric symptoms differ in their assumptions regarding what those symptoms are. Though empirically defensible, this is contention, needs to be contextualized by considering *when* (i.e., under what circumstances) symptom measurement heterogeneity can become problematic. Without this contextualization, it risks appearing overly pessimistic, as it might suggest that symptom measurement heterogeneity is inherently detrimental and, in any case, an undesirable feature of psychiatry. In what follows I refine my contention by briefly considering how the heterogeneity of *construct operationalization*^3^ is not necessarily a negative attribute of symptom measurement in psychiatry. For this, I turn to a key remark from the philosophy of measurement debate, namely, that such heterogeneity can be justified by the fact that different measures of (purportedly) the same thing often serve different purposes in different contexts. If so, then it is reasonable in each case to focus on the aspects of the thing under study that are most relevant to assess for a particular end. This point has been extensively discussed by Anna Alexandrova in several places (2014, 2017, 2021) including her philosophy book on the science of well-being. I shall now turn to her formulation of it, as found in her book.

#### Construct pluralism: lessons from well-being studies

The study of well-being spans across several scientific disciplines, including economics, sociology, psychology, and medicine. Each of these disciplines adopts a different approach to defining and measuring well-being. As a result, the investigative landscape of well-being studies is best characterized by what Alexandrova calls *construct pluralism*. This term refers to the diversity of operationalizations and measurements stemming from the study of complex constructs, like well-being, across different scientific domains. The main methodological tasks that construct pluralism presents us with is answering to the following question: which of the many things called ‘well-being’ in the sciences is the correct construct to use and for which purpose? (2017, p. XXXV). This question presupposes what she later on calls *variantism* (ibid, p. 40), which comes in a strong and a moderate version. According to the former, “there is not one concept [of well-being] and there is not one thing that many concepts refer to” (ibid, p. 44) rather, well-being can be understood and operationalized differently depending on the context and purpose of the measurement. According to the latter, “it may be that there is a single correct theory of well-being, but for the purpose of construct justification we need not assume one” (ibid.); instead, it suffices to rely on local arguments that justify the use of different constructs of well-being for different goals in a context-sensitive manner. In this article, I treat the two versions of variantism indistinctly because both share the core tenet that well-being can be understood and operationalized variously depending on the goals pursued in specific contexts. Moreover, I take the difference between construct pluralism and variantism to be an attitudinal one, that is, while the former describes a feature of the science of well-being, the latter tells us that a contextual understanding of well-being is the way to go.

In discussing the measurability of well-being, Alexandrova argues that an all-encompassing conception of well-being (i.e., one that requires a complete aggregation of all the relevant goods for an agent in a way that respects their history and personal traits) might not be measurable. However, this does not mean that more contextual and less demanding conceptions of it cannot be measured (ibid, p. 117). Constructs of well-being are diverse, among other things, because they pick out different aspects, causes or correlates from the numerous factors that bring about well-being (ibid, p. 46). Measures of well-being may then vary accordingly. Despite this diversity, these context-tailored constructs can still be thought of as part of the same overarching concept by having a shared core across disciplines, namely, their goal of improving or understanding aspects of human flourishing.

I am sympathetic to Alexandrova’s variantist stance with regard to well-being. I also believe that her analysis likely extends to other study-objects whose inquiry spans across several scientific disciplines and subdisciplines. In this article, I confine my analysis to exploring the applicability of the variantist stance to the case of symptoms in order to elucidate under what circumstances symptom measurement heterogeneity is problematic. To be sure, I do not assume that symptom measurement heterogeneity is inherently problematic. My contention is rather that it *can* be problematic just like diagnostic heterogeneity can be so, and this remains greatly overlooked by the transdiagnostic approach. That said, I now suggest that the difference between variantism and symptom measurement heterogeneity is mainly one of scope and emphasis. Put simply, variantism is a stance that embraces construct diversity across scientific fields and contexts, based on the diversity of goals of measurement practices within each domain. Symptom measurement heterogeneity, on the other hand, focuses on a more restricted context (i.e., symptom measurement for clinical and research purposes) within the domain of psychiatry. Still, it could be argued that *within* the clinical and the research contexts in psychiatry, both of which are broad, different measures can serve different purposes. Each of these purposes may locally justify a different operationalization of a construct such as anhedonia depending on the particular aspect thereof that is relevant for the purpose at stake. Here is where the difference of emphasis comes in. Acknowledging that construct diversity can be locally justified in a context-sensitive manner, does not imply that there is no context where such diversity (or heterogeneity) can turn out to be detrimental. A crucial element of the variantist stance is the requirement of *justification* for operationalizing a construct in a locally appropriate way. Part of my contention is that such kind of justification may lack in contexts where the received view of symptoms is adopted rather uncritically, as in transdiagnostic research. The nature and amount of justification needed for “construct-tailoring” in each case will likely vary with context and purpose too. Moreover, in some cases it may not be self-evident to researchers what counts as sufficient justification, which may lead to disagreement. By the same token, the extent to which symptom measurement heterogeneity is detrimental, or at least problematic, will likely vary with context and purpose. In any case, the assumption that symptoms are straightforward things whose measures are interchangeable disregards the fact that *some* kind of local justification may be needed in the first place.

Providing an account of local justification in Alexandrova’s sense lies beyond the scope of this article. Nevertheless, something can be said about *when* symptom measurement heterogeneity is problematic. I distinguish the following two non-exhaustive scenarios. Firstly, symptom measurement heterogeneity can be problematic in cases where a local justification for the use of one or several specific symptom measures is needed and not provided, i.e., when the reasons for using x or y measures are not stated in contexts where the choice to be made is not self-evident. This encompasses, for instance, cases where the interchangeability of symptom measures is assumed in a context-negligent way. In referring to the ‘use’ of a specific symptom measure I include the use of the data generated with it. Thus, failing to provide justification in a specific research context for extrapolating the results obtained with a specific measure of anhedonia to anhedonia in general can also be a problematic case of symptom measurement heterogeneity. A second, related scenario is one where symptom measurement heterogeneity gives rise to mutually inconsistent datasets or datasets that diverge in some context-relevant way. This happens, for instance, if the scientific literature provides ambiguous or even conflicting evaluations of the clinical efficacy of a psychiatric drug on a specific symptom. In the lack of robust datasets, the revision processes of psychiatric classifications might be slowed down and tend to favor a *status quo* where symptom concepts remain unchanged. Certainly, not all possible inconsistencies between different datasets are equally critical across contexts. For example, inconsistencies between datasets measuring sleep disturbance using different self-report tools may not be problematic in certain contexts, such as general population surveys aimed at estimating the prevalence of sleep issues. Different tools might yield somewhat different prevalence rates, but these discrepancies would not impede the observation of population-level trends or correlations, such as associations between sleep disturbance and stress levels. This shows that local arguments can (and should) be provided in contexts where diverging datasets are used in order to justify why such a divergence is or not relevant for the purpose at stake. Failing to do so when needed, would constitute a case of problematic symptom measurement heterogeneity.

### Some insights into diagnostic validity

Throughout this article I have used the term diagnostic validity as referring to the accuracy with which a diagnostic category leads to the identification of a homogeneous group of clinical instances of a specific mental disorder. Symptom measurement heterogeneity, I argued, can increase the variability in what is being measured and thus lower the general homogeneity of the groups diagnosed under the same category. This can be critical in cases where the symptom under scrutiny is a core symptom, as in the case of anhedonia with respect to MDD. But putting this general comment aside, diagnostic validity as here defined leaves open two interwoven questions. Firstly, by what standards do researchers determine the required level of homogeneity that groups of co-diagnosed individuals should have? And second, in what respect or, more broadly, at what level of description should a group of individuals be homogeneous to be considered instances of the same mental disorder? These are difficult questions for which I have no definite answer. Different researchers and clinicians may respond differently depending on what they consider to be the “right” causal story of a disorder as well as the most promising *locus of treatment* for it. However, in all likelihood, answers will invoke a multiplicity of factors at various levels of description (see Kendler et al., 2020 for an excellent volume on the topic). An important remark needs to be made here, one that has already been stated in some way by several philosophers (Zachar, 2020; Bortolotti & Broome, 2009; Ratcliffe, 2009). Regardless what individual researchers may favor as the “right” causal story of a mental disorder, referring to symptoms is ultimately unavoidable. The *indispensability* of symptom-reference stems from several sources. Most clearly, the experience of symptoms (i.e., instances thereof) is what brings people to the clinic in the first place, but also, the effectiveness of most treatments is judged in terms of their capacity to ameliorate symptoms and thus improve functionality or quality of life. Furthermore, methods of validation such as those proposed by Robins & Guze’s, 1970 paper often rely on the observation of symptoms (e.g., in clinical description and differential diagnosis). It follows from this that the diagnostic validity of psychiatric categories can hardly be assessed with independence of their capacity to accurately capture and organize symptoms. Even if psychiatry’s efforts focused on exploring the neural correlates of mental disorders to develop psychiatric drugs, any attempt to do so will require a sharp understanding of the relevant experiences and behaviors (i.e., the symptoms). But how sharp is sharp enough? If symptom measurement heterogeneity is a real issue, the following question persists: how homogenous should the group of instances of a *symptom* be in order to avoid the heterogeneity found at the diagnostic level?

There is arguably no single standard by which researchers determine what degree of heterogeneity is admissible across a co-diagnosed population such that the corresponding diagnostic category can be deemed diagnostically valid. I think this can be said of symptoms too. There is no acontextual way of saying when the array of experiences and behaviors comprised under the term “anhedonia” is too heterogeneous, which is why local arguments matter. More generally, judgements of heterogeneity in psychiatry are not made in a *vacuum*; they too presuppose a context and a purpose. From a general perspective, such purpose is essentially the identification of promising targets for clinical intervention. However, in psychiatry, clinical intervention and treatment come in many flavors; as a result, there is no single standard to judge admissible degrees of heterogeneity. In principle, lumping together anhedonic patients with schizophrenia *and* anhedonic patients with MDD might work for certain clinical effects but not for others. This does not preclude the judgement that the degree of heterogeneity associated a diagnostic category is, on balance, suboptimal.

Groups of co-diagnosed individuals are deemed heterogeneous not simply by virtue of having different symptom-profiles (in a trivial sense, they will always be different), but mainly, because such groupings generally do not yield good targets for clinical intervention or, at least, not the targets transdiagnostic researchers are interested in. Similarly, whether symptom terms are heterogeneous by virtue of being multiply operationalized will partly depend, in addition to the considerations of the previous subsection, on whether they pin down something of clinical use. Importantly, this does not amount to say that diagnostic validity and clinical utility are the same thing; rather, it means that diagnostic validity (as defined in this article) can hardly be assessed without considering the clinical-pragmatic dimension of diagnosis and measurement. In practice, this involves confronting heterogeneity-claims about symptom-measurement or diagnosis with the following question: too heterogeneous *for what*? While not a rule of thumb, the answer to this question will likely evoke some reference to potentially fruitful targets for clinical intervention or envisaged treatment options.

Consequently, an eventual reduction of symptom measurement heterogeneity in transdiagnostic research is not per se a solution to the lack of diagnostic validity. Such reduction, when needed, must be guided by a good understanding of the degree and type of heterogeneity that is admissible given a specific clinical or research purpose. If successful, this might increase transdiagnostic research’s chance at stumbling upon clinically actionable constructs within more local scenarios. All this, I argued, would presuppose the abandonment of the received view of symptoms.

## Conclusion

In this article, I have argued that the received view of symptoms readily adopted by some transdiagnostic research initiatives—such as RDoC—is unwarranted. Some of these initiatives promote the abandonment of the DSM categories emphasizing their lack of diagnostic validity while still heavily relying on traditional psychiatric symptoms under the assumption that they do not pose similar problems. In particular, the received view of symptoms presumes that symptom constructs are not problematically infused with the DSM categories, and also, that they do not differ from each other in potentially relevant ways within the clinical and research contexts. I used a case study from the history of the measurement of anhedonia to show that psychiatric symptoms are not neutral vis-à-vis the DSM categories. In fact, some measures of anhedonia were developed with the explicit goal of discriminating between different diagnostic categories. Based on the same case study, I showed that different instruments of purportedly the same thing often presuppose constructs that differ from one another with regard to what exactly the thing being measured is. Crucially, those differences need not be underpinned by different theories about the etiology of symptoms; instead, they may take the form of diverging working assumptions embedded in the constructs of different instruments. Stemming from this observation, I claimed that in addition to the diagnostic heterogeneity widely documented in the psychiatry literature, there is also symptom measurement heterogeneity. By considering Alexandrova’s variantist stance, I showed that the heterogeneity discussed in this article is not inherently problematic or unjustifiable, but it can become so in certain contexts, such as those where local arguments are missing. Finally, I argued that the lack of diagnostic validity is likely linked to symptom measurement heterogeneity, but addressing this may be more effective through context-specific and purpose-driven approaches rather than simply reducing such heterogeneity.

## Footnotes

1 Although comorbidity often complicates diagnosis, it is not always problematic. In the clinical context, some degree of comorbidity among certain diagnoses is often accepted and potentially useful. Sometimes comorbid conditions can be addressed through integrated treatments that tackle multiple issues at once. For example, therapies that address both PTSD and substance use, such as trauma-focused therapies, have been developed specifically because of the high comorbidity between these conditions.

2 As he notes in his article, Fellowes uses the term ‘symptoms’ to refer to both signs and symptoms in psychiatry.

3 Here I used the term *construct* as being relative to a specific measurement tool, i.e., a symptom measure presupposes a symptom construct. Alexandrova’s use of the term *construct* corresponds to my use of the term *concept*. Other things being equal, her point can be readily applied to the context of this article.
